
JOURNAL INFORMATION
==============================
NLM Title Abbreviation: ScientificWorldJournal
Journal ID: ScientificWorldJournal
NLM Journal ID: 101131163
Journal ID: TSWJ

The Scientific World Journal

ISSN: 2356-6140
EISSN: 1537-744X
Publisher: Wiley

ARTICLE INFORMATION
==============================
PMCID: PMC5823116
PMID: 19347233
DOI: 10.1100/tsw.2009.28
Article ID: 656471
Article version: 1
Subjects: Directions in Science

Potential of Small Molecule Protein Tyrosine Kinase Inhibitors as Immunomodulators and Inhibitors of the Development of Diabetes

Little Peter J. 1 2 *
Cohen Neale 3
Morahan Grant 4
1 Diabetes and Cell Biology Laboratory, BakerIDI Heart and Diabetes Institute, Melbourne, VIC 3004, Australia
2 Medicine and Immunology Departments, Central and Eastern Clinical School, Alfred Hospital, Monash University, Melbourne, VIC 3004, Australia
3 Clinical Services Division, BakerIDI Heart and Diabetes Institute, Melbourne,VIC 3004, Australia
4 Centre for Diabetes Research, Western Australian Institute for Medical Research, 50 Murray Street, Perth WA 6000, Australia
*Peter J. Little: peter.little@bakeridi.edu.auAcademic Editor: Lawrence A. Frohman

Publication date: 2009
Electronic publication date: 2009 Mar 31
Volume: 9
First page: 224
Last page: 228
Received 2009 Jan 16; Revised 2009 Mar 17; Accepted 2009 Mar 19
Copyright: Copyright © 2009 Peter J. Little et al.
Copyright year: 2009
License: This is an open access article distributed under the Creative Commons Attribution License, which permits unrestricted use, distribution, and reproduction in any medium, provided the original work is properly cited.
License URL: https://creativecommons.org/licenses/by/3.0/

Keywords: Type 1 diabetes, Type 2 diabetes, imatinib, autoimmunity, NOD mouse

==============================
Directions in Science
*TheScientificWorldJOURNAL* (2009) 9, 224–228
ISSN 1537-744X; DOI 10.1100/tsw.2009.28

TheScientificWorldJOURNAL
www.thescientificworld.com

### Potential of Small Molecule Protein Tyrosine Kinase Inhibitors as Immunomodulators and Inhibitors of the Development of Diabetes

Peter J. Little[1,2,*], Neale Cohen[3], and Grant Morahan[4]

*[1]Diabetes and Cell Biology Laboratory, BakerIDI Heart and Diabetes Institute, Melbourne, VIC, 3004, Australia; [2]Medicine and Immunology Departments, Central and Eastern Clinical School, Alfred Hospital, Monash University, Melbourne, VIC, 3004, Australia; [3]Clinical Services Division, BakerIDI Heart and Diabetes Institute, Melbourne, VIC, 3004, Australia; [4]Centre for Diabetes Research, Western Australian Institute for Medical Research, 50 Murray Street, Perth WA 6000, Australia*

**E-mail:** peter.little@bakeridi.edu.au; neale.cohen@bakeridi.edu.au; gem@waimr.uwa.edu.au

*Received January 16, 2009; Revised March 17, 2009; Accepted March 19, 2009; Published March 31, 2009*

---

**KEYWORDS:** Type 1 diabetes, Type 2 diabetes, imatinib, autoimmunity, NOD mouse

---

Type 1 diabetes (T1D) (previously insulin-dependent or juvenile-onset diabetes) is one of the two major forms of human diabetes[1]. T1D is caused by an autoimmune process involving T cells, B cells, monocyte macrophages, and dendritic cells, with the major pathology being the development of destructive T-cell infiltration of insulin-producing β cells in the pancreas. Disease occurs from the unfavorable balance of susceptibility and resistance genes, and is thought to have an unidentified environmental trigger. The β-cell destruction results in an absolute deficiency of insulin with chronic hyperglycemia. Medical treatment is insulin administration for life and therapeutic attention to comorbidities, such as hyperlipidemia and hypertension. The pathological consequences of the diabetic milieu, predominantly hyperglycemia, are known as "complications", and these include vasculopathies of increased and premature strokes, heart attacks, limb amputations, kidney disease, and blindness. Evidence suggests that T1D might also be associated with peripheral insulin resistance[2,3], the precipitating factor in obesity-induced Type 2 diabetes (T2D), and there might be closer associations between these two most prominent forms of diabetes than previously recognized.

T1D is a devastating disease because it can commence at a very young age and requires lifelong multiple daily insulin injections and constant invasive monitoring of blood glucose levels. T1D commences mostly with rapid onset in children and young adults. The prevalence of T1D is increasing, especially in older subjects, but the reasons for this are unknown.

Most of the advances in the understanding of this disease over the last 3 decades have been from an immunological and genetic perspective. In the 1980s, physician scientists, immunologists, and diabetologists offered the hope that T1D would be cured by the end of the 20th century. Despite extensive research and many immunologically based trials, this outcome did not eventuate and there remains no therapeutic intervention for the prevention or cure of this disease to this day.

*Corresponding author.
©2009 with author.
Published by TheScientificWorld; www.thescientificworld.com

In humans, it is possible to identify a prediabetic state characterized by the presence of autoantibodies to islet cell molecules. This offers an opportunity for intervention before disease onset. Strategies in the preclinical phase involving parenteral, oral, or intranasal insulin showed no effect on the rate of occurrence of clinical diabetes[4], but further trials are continuing with modified intervention strategies. The most successful agent currently is the anti–T-lymphocyte approach utilizing anti–CD3 antibodies, but the duration of action is not confirmed[5]. Agents that have been through trials at the time of diagnosis include cyclosporine A, imuran, corticosteroids, BCG vaccine, and nicotinamide, but these have had no lasting effect so are not viable strategies for the secondary prevention of T1D.

If the above approaches using antibodies and proteins may be considered as a bioimmunological approach, then what might be termed a chemicoimmunological approach has recently been described in two reports of the successful prevention and even reversal of diabetes (hyperglycemia) in established animal models of human T1D[6,7]. Both studies utilized the nonobese diabetic (NOD) mouse, which is considered to be phenotypically and genetically a suitable model of T1D[7,8]. Most female NOD mice spontaneously develop hyperglycemia by 12–14 weeks of age and the disease process shares many similarities with T1D.

Small-molecule protein tyrosine kinase inhibitors (PTKIs) can prevent or even reverse the development of diabetes in the NOD mouse[6,7]. The action of the PTKIs may well be mediated via actions on the immune system and such a simple treatment, especially if well tolerated and of low toxicity, is highly appealing and would provide a major breakthrough.

The prototypical compound that has been described is the 2-phenylaminopyrimidine derivative imatinib (formally STI571 and CGP57148B). Imatinib inhibits only a small number of PTKs — c-Abl, c-Arg, PDGFR, and c-Kit. It has been widely tested and is known not to inhibit a very wide range of other kinases[9]. Imatinib was strategically developed as a specific "smart" inhibitor of the c-Abl kinase activity of the c-Abl-Bcr (Philadelphia chromosome) in chronic myeloid leukemia (CML) and subsequently as an inhibitor of c-Kit in c-Kit–expressing gastrointestinal stromal tumors[9]. Patients with a common form of CML respond positively to imatinib and tolerate the drug very well.

Imatinib also has immunomodulatory activity. Imatinib impairs adaptive and innate immune functions of T cells, dendritic cells, and monocyte macrophages. Imatinib has an anti-inflammatory activity by decreasing the production of TNFα by macrophages[10]. Imatinib has also shown activity in animal models of autoimmunity. It prevents and treats murine collagen-induced arthritis, where it does so by inhibiting a range of signaling pathways, and it prevents autoimmune nephritis in a mouse model of lupus, in this case by inhibiting PDGF signaling[11]. In case reports, imatinib has been shown to inhibit autoimmune disease in humans, including activity against rheumatoid arthritis, psoriasis, Crohn's disease, and diabetes (see later).

Hagerkvist et al. described the protective effect of imatinib on two models of hyperglycemia in the mouse[6]. As well as the NOD model, these authors injected male NMRI mice with streptozotocin (STZ), a treatment that results in hyperglycemia due to direct toxicity of STZ on the β cells of the pancreas. Imatinib protected against the development of hyperglycemia in both models[6]. *In vitro*, imatinib protected β cells against cell death induced by cytokines and STZ. This effect of imatinib *in vitro* was mimicked by siRNA to c-Abl; thus it was concluded that c-Abl kinase was the likely target in the *in vivo* experiments[6]. c-Abl is a good candidate because it is activated when cells are damaged, leading to cell cycle arrest and apoptosis. c-Abl phosphorylation promotes downstream activation of stress effector pathways, such as JNK and p38 MAP kinases.

Louvet et al.[7] reported that imatinib treatment prevents the development of hyperglycemia in NOD mice and reverses established diabetes. The response is partially mimicked by a soluble form of PDGF receptor, but not agents targeting other imatinib targets, so it was concluded that the action occurred via PDGF receptor inhibition[7]. Intriguingly, imatinib did not significantly reduce the leukocyte infiltration of the pancreas.

These effects of imatinib on T1D follow on from reports of its efficacy in T2D. For example, a case was reported of a nonobese 70-year-old woman with insulin-requiring T2D of 8 years standing, who was treated for CML with imatinib (400 mg/day). Hematological remission was achieved 2 months after commencing therapy and, concomitantly, the patient's plasma glucose and insulin levels declined to a level where she did not require insulin and the diagnosis of diabetes was no longer applicable[12]. Her diabetes-free status was confirmed after 1 year[12].

To validate imatinib as a treatment for T1D, it is necessary to know the target of drug action. The two papers discussed above suggest different targets: c-Abl kinase[3] and the PDGF receptor[4]. We have had extensive experience with imatinib and its derivatives[13] in the setting of the prevention of atherosclerosis. From our data and experience, we speculate that the ultimate target of imatinib in some non-CML settings may be a novel PTK and it is possible that this may be the case for the prevention of hyperglycemia in the mouse models.

A considerable amount of further work is required to identify the target(s) of imatinib's action so that better therapeutic agents can be developed. It is necessary to confirm the mechanism of action of imatinib, in animals and ultimately in humans, so that a greater understanding of the disease process and its progression can occur, and also to identify any patient subgroups that may be sensitive or resistant to imatinib intervention.

Although these results are very promising, there is a major caveat: the experimental results with imatinib and diabetes were obtained in the NOD mouse model — thus, where earlier successful immunologically based studies in NOD mice have not transferred to the clinical situation, the initial data with imatinib may also be a false dawn. The value of this drug for treating human T1D can only be judged from suitable clinical trials, and it is hoped that the preliminary animal data combined with the fact that imatinib is an existing therapeutic agent can provide sufficient impetus for trials to occur as soon as ethical and safety concerns are satisfied.

Although this article focuses on the role of PTKIs in autoimmune diabetes, it should be recognized that there is also evidence for a role for these agents in ameliorating insulin resistance and T2D. Imatinib treatment is associated with regression of T2D in some[12,14], but not all, studies[15]. Most recently, it has been shown that imatinib reverses hyperglycemia in the *db/db* mouse model of insulin resistance and T2D[16]. In this study, insulin resistance is related to the expression of a family of genes related to endoplasmic reticulum (ER) stress[17], the unfolded protein response, in multiple organs[16]. Imatinib treatment reduces the expression of these genes and also directly inhibits expression of these genes in a model cell line, indicating that the action is direct[16]. ER stress is thought to be downstream of c-Abl, the target of imatinib in CML and also the purported target in insulin resistance. Imatinib counteracts high-fat-fed peripheral insulin resistance in rats at 50 mg/kg, but not at 100 mg/kg, where the latter concentration is associated with a variety of toxicities[18]. Imatinib treatment specifically improves insulin sensitivity in nondiabetic subjects with CML who, interestingly, had significant insulin resistance[19]. Six patients given imatinib for CML had insulin sensitivity assessed by euglycemic clamp, and all subjects treated with 400 mg/day imatinib for 3 months achieved a substantial improvement in their primary disease and all demonstrated significantly higher insulin sensitivity[19]. Actions in humans are not restricted to imatinib because sunitinib (SU11248) treatment for 4 weeks lowered blood glucose by almost 2 m*M* on average in all 19 subjects treated with sunitinib for metastatic renal cell carcinoma[20]. However, the question of the role of imatinib in reversing hyperglycemia of T2D is certainly not resolved because an evaluation of the records of 164 imatinib-treated patients at the Mayo Clinic, of whom seven had diabetes, found no improvement in the glycemic status of these patients following an average 16 months therapy with imatinib and two patients developed diabetes in the study period[15].

If β-cell destruction and peripheral insulin resistance contribute to both T1D and T2D, then the reports that imatinib and related molecules can address both of these pathologies is very exciting. This class of PTKIs, at least in theory, has the potential for a major impact on hyperglycemia in multiple forms of diabetes. Imatinib's protective actions on cardiovascular and renal disease[21,22] further points to the potential of PTKIs and raises the need to investigate these agents fully.

In conclusion, the initial data showing that a small-molecule PTKI can prevent or even reverse the development of diabetes in an established animal model provide an exciting opportunity for a major therapeutic advance in an area that is currently devoid of therapies. If imatinib, perhaps at low doses, could be used to treat children with recently diagnosed T1D, or even those with known risk factors, such as a family history of the disease and/or genetic and immunological biomarkers, then even if it was only successful in preventing development of the disease in a small proportion of subjects, it would still represent a major breakthrough and advance. Of course, for those millions of people in the world with existing T1D, the biggest potential news is the indication from the animal study that T1D can be reversed and this possibility is one of the most exciting to arise in this area for many years. Finally, results in humans of the successful reversal of T2D by imatinib, and the animal and human data showing the amelioration of insulin resistance, even allows some possibility of protective activity in that condition that affects approximately ten times more people than does T1D.

### ACKNOWLEDGMENTS

We acknowledge expert guidance from Prof. Peter Colman, Department of Diabetes and Endocrinology, Royal Melbourne Hospital, in relation to diabetes prevention trials and for commenting on the paper. The authors acknowledge research support from the Diabetes Australia Research Trust and National Health and Medical Research Council of Australia.

### REFERENCES

1. Colman, P.G., Thomas, D.W., Zimmet, P.Z., Welborn, T.A., Garcia-Webb, P., and Moore, M.P. (1999) New classification and criteria for diagnosis of diabetes mellitus. Position Statement from the Australian Diabetes Society, New Zealand Society for the Study of Diabetes, Royal College of Pathologists of Australasia and Australasian Association of Clinical Biochemists. *Med. J. Aust.* **170,** 375–378.
2. Fourlanos, S., Narendran, P., Byrnes, G.B., Colman, P.G., and Harrison, L.C. (2004) Insulin resistance is a risk factor for progression to type 1 diabetes. *Diabetologia* **47,** 1661–1667.
3. Couper, J.J., Beresford, S., Hirte, C., Baghurst, P.A., Pollard, A., Tait, B.D., Harrison, L.C., and Colman, P.G. (2009) Weight gain in early life predicts risk of islet autoimmunity in children with a first-degree relative with type 1 diabetes. *Diabetes Care* **32,** 94–99.
4. Nanto-Salonen, K., Kupila, A., Simell, S., Siljander, H., Salonsaari, T., Hekkala, A., Korhonen, S., Erkkola, R., Sipila, J.I., Haavisto, L., Siltala, M., Tuominen, J., Hakalax, J., Hyoty, H., Ilonen, J., Veijola, R., Simell, T., Knip, M., and Simell, O. (2008) Nasal insulin to prevent type 1 diabetes in children with HLA genotypes and autoantibodies conferring increased risk of disease: a double-blind, randomised controlled trial. *Lancet* **372,** 1746–1755.
5. Herold, K.C., Gitelman, S.E., Masharani, U., Hagopian, W., Bisikirska, B., Donaldson, D., Rother, K., Diamond, B., Harlan, D.M., and Bluestone, J.A. (2005) A single course of anti-CD3 monoclonal antibody hOKT3gamma1(Ala-Ala) results in improvement in C-peptide responses and clinical parameters for at least 2 years after onset of type 1 diabetes. *Diabetes* **54,** 1763–1769.
6. Hagerkvist, R., Sandler, S., Mokhtari, D., and Welsh, N. (2007) Amelioration of diabetes by imatinib mesylate (Gleevec): role of beta-cell NF-kappaB activation and anti-apoptotic preconditioning. *FASEB J.* **21,** 618–628.
7. Louvet, C., Szot, G.L., Lang, J., Lee, M.R., Martinier, N., Bollag, G., Zhu, S., Weiss, A., and Bluestone, J.A. (2008) Tyrosine kinase inhibitors reverse type 1 diabetes in nonobese diabetic mice. *Proc. Natl. Acad. Sci. U. S. A.* **105,** 18895–18900.
8. Leiter, E.H. (2005) Nonobese diabetic mice and the genetics of diabetes susceptibility. *Curr. Diab. Rep.* **5,** 141–148.
9. Traxler, P., Bold, G., Buchdunger, E., Caravatti, G., Furet, P., Manley, P., O'Reilly, T., Wood, J., and Zimmermann, J. (2001) Tyrosine kinase inhibitors: from rational design to clinical trials. *Med. Res. Rev.* **21,** 499–512.
10. Wolf, A.M., Wolf, D., Rumpold, H., Ludwiczek, S., Enrich, B., Gastl, G., Weiss, G., and Tilg, H. (2005) The kinase inhibitor imatinib mesylate inhibits TNF-{alpha} production in vitro and prevents TNF-dependent acute hepatic inflammation. *Proc. Natl. Acad. Sci. U. S. A.* **102,** 13622–13627.
11. Paniagua, R.T., Sharpe, O., Ho, P.P., Chan, S.M., Chang, A., Higgins, J.P., Tomooka, B.H., Thomas, F.M., Song, J.J., Goodman, S.B., Lee, D.M., Genovese, M.C., Utz, P.J., Steinman, L., and Robinson, W.H. (2006) Selective tyrosine kinase inhibition by imatinib mesylate for the treatment of autoimmune arthritis. *J. Clin. Invest.* **116,** 2633–2642.
12. Veneri, D., Franchini, M., and Bonora, E. (2005) Imatinib and regression of type 2 diabetes. *N. Engl. J. Med.* **352,** 1049–1050.
13. Ballinger, M.L., Osman, N., Wilks, A.F., Su, S., Burns, C.J., Bu, X., and Little, P.J. (2008) Pyrido-pyrimidine derivative CYC10424 inhibits glycosaminoglycan changes on vascular smooth muscle-derived proteoglycans and reduces lipoprotein binding. *J. Cardiovasc. Pharmacol.* **52,** 403–412.
14. Breccia, M., Muscaritoli, M., Aversa, Z., Mandelli, F., and Alimena, G. (2004) Imatinib mesylate may improve fasting blood glucose in diabetic Ph+ chronic myelogenous leukemia patients responsive to treatment. *J. Clin. Oncol.* **22,** 4653–4655.
15. Dingli, D., Wolf, R.C., and Vella, A. (2007) Imatinib and type 2 diabetes. *Endocr. Pract.* **13,** 126–130.
16. Han, M.S., Chung, K.W., Cheon, H.G., Rhee, S.D., Yoon, C.H., Lee, M.K., Kim, K.W., and Lee, M.S. (2009) Imatinib mesylate reduces endoplasmic reticulum stress and induces remission of diabetes in db/db mice. *Diabetes* **58,** 329–336.
17. Xu, C., Bailly-Maitre, B., and Reed, J.C. (2005) Endoplasmic reticulum stress: cell life and death decisions. *J. Clin. Invest*. **115,** 2656–2664.
18. Hagerkvist, R., Jansson, L., and Welsh, N. (2008) Imatinib mesylate improves insulin sensitivity and glucose disposal rates in rats fed a high-fat diet. *Clin. Sci. (Lond.)* **114,** 65–71.
19. Tsapas, A., Vlachaki, E., Sarigianni, M., Klonizakis, F., and Paletas, K. (2008) Restoration of insulin sensitivity following treatment with imatinib mesylate (Gleevec) in non-diabetic patients with chronic myelogenic leukemia (CML). *Leuk. Res*. **32,** 674–675.
20. Billemont, B., Medioni, J., Taillade, L., Helley, D., Meric, J.B., Rixe, O., and Oudard, S. (2008) Blood glucose levels in patients with metastatic renal cell carcinoma treated with sunitinib. *Br. J. Cancer* **99,** 1380–1382.
21. Lassila, M., Allen, T.J., Cao, Z., Thallas, V., Jandeleit-Dahm, K.A., Candido, R., and Cooper, M.E. (2004) Imatinib attenuates diabetes-associated atherosclerosis. *Arterioscler. Thromb. Vasc. Biol.* **24(5),** 935–942.
22. Wang, S., Wilkes, M.C., Leof, E.B., and Hirschberg, R. (2005) Imatinib mesylate blocks a non-Smad TGF-beta pathway and reduces renal fibrogenesis in vivo. *FASEB J*. **19,** 1–11.

**This article should be cited as follows:**

Little, P.J., Cohen, N., and Morahan, G. (2009) Potential of small molecule protein tyrosine kinase inhibitors as immunomodulators and inhibitors of the development of diabetes. *TheScientificWorldJOURNAL* **9**, 224–228. DOI 10.1100/tsw.2009.28.
